# The interactions between vitamin D and neurofilament light chain levels on cognitive domains in bipolar disorder

**DOI:** 10.1192/bjo.2022.608

**Published:** 2022-11-28

**Authors:** Wen-Yin Chen, Ming-Chyi Huang, Chih Chiang Chiu, Ying-Chih Cheng, Chian-Jue Kuo, Po-Yu Chen, Po-Hsiu Kuo

**Affiliations:** Department of Psychiatry, Taipei City Psychiatric Center, Taipei City Hospital, Songde branch, Taiwan; and Institute of Epidemiology and Preventive Medicine, College of Public Health, National Taiwan University, Taiwan; Department of Psychiatry, Taipei City Psychiatric Center, Taipei City Hospital, Songde branch, Taiwan; and Department of Psychiatry, School of Medicine, College of Medicine, Taipei Medical University, Taiwan; Institute of Epidemiology and Preventive Medicine, College of Public Health, National Taiwan University, Taiwan; and Department of Psychiatry, China Medical University Hsinchu Hospital, China Medical University, Taiwan; Institute of Epidemiology and Preventive Medicine, College of Public Health, National Taiwan University, Taiwan; Department of Public Health, College of Public Health, National Taiwan University, Taiwan; Department of Psychiatry, National Taiwan University Hospital, Taiwan; and Psychiatric Research Center, Wan Fang Hospital, Taipei Medical University, Taiwan

**Keywords:** Brief assessment of cognition in affective disorders, bipolar disorders, vitamin D, neurofilament light chain, cognition

## Abstract

**Background:**

Bipolar disorder is a chronic mental disorder related to cognitive deficits. Low serum vitamin D levels are significantly associated with compromised cognition in neuropsychiatric disorders. Although patients with bipolar disorder frequently exhibit hypovitaminosis D, the association between vitamin D and cognition in bipolar disorder, and their neuroaxonal integrity, is unclear.

**Aims:**

To investigate the interaction effects between vitamin D and neurofilament light chain (NfL) levels on cognitive domains in bipolar disorder.

**Method:**

Serum vitamin D and NfL levels were determined in 100 euthymic patients with bipolar disorder in a cross-sectional study. Cognitive function was measured with the Brief Assessment of Cognition in Affective Disorders. We stratified by age groups and used general linear models to identify associations between vitamin D and NfL levels and their interaction effects on cognitive domains.

**Results:**

The mean vitamin D and NfL levels were 16.46 ng/nL and 11.10 pg/mL, respectively; 72% of patients were vitamin D deficient. In the older group, more frequent hospital admissions and lower physical activity were identified in the group with versus without vitamin D deficiency. The age-modified interaction effect of vitamin D and NfL was associated with composite neurocognitive scores and verbal fluency in both age groups, and with processing speed domain in the younger group.

**Conclusions:**

We observed a high vitamin D deficiency prevalence in bipolar disorder. We identified the interaction of vitamin D and NfL on cognitive domains, and the effect was modified by age. Longitudinal or randomised controlled studies enrolling patients with various illness durations and mood statuses are required to validate our findings.

Bipolar disorder is a major psychiatric illness characterised by its early onset, severity and alternating mood episodes.^1^ A considerable proportion of bipolar disorder cases have been significantly associated with cognitive deficits, although to varying degrees, even when the bipolar disorder is in remittance.^2^ Studies have also suggested that patients with bipolar disorder can experience accelerated cognitive ageing, which presents as a steeper decline in function.^3,4^ Cognitive impairment in patients with bipolar disorder may worsen as the disease progresses, which may negatively affect patient recovery.^5^ In addition, age can dynamically, neurodevelopmentally and neuroprogressively interact with the neuropathology of bipolar disorder.^6^ Although cognitive dysfunction has increasingly been recognised as a key symptom in patients with bipolar disorder, the biomarkers related to cognitive dysfunction have rarely been investigated, especially in different age groups of patients with bipolar disorder.

Evidence suggests that vitamin D deficiency may be a key marker of cognitive decline.^7^ Moreover, higher vitamin D levels and higher vitamin D supplement intake have been associated with a slower cognitive decline.^8,9^ This indicates that vitamin D may play an essential role in brain health and function, and may exert various neuroprotective effects on numerous brain areas.^10^ A survey revealed that more than a third of patients with severe mental disorders, including bipolar disorder, were deficient in vitamin D.^11^ In addition, clinical studies suggested that vitamin D may play a role in the pathophysiology or cognitive profile of bipolar disorder. For example, a negative and moderate correlation was identified between vitamin D levels and mania severity or disease severity in bipolar disorder.^12^ Polymorphism in the vitamin D receptor gene was associated with lower expression of the dopamine D1 receptor gene and may contribute to bipolar disorder development.^13^ Furthermore, vitamin D deficiency has been associated with poor cognitive functioning in individuals experiencing major depressive episodes and in psychotic disorders, including psychotic bipolar disorder.^14,15^ An image study suggested a significant and positive association between vitamin D and intracranial volume in psychotic bipolar disorder.^16^ In summary, although bipolar disorder has been reported to be associated with altered cognitive functioning and hypovitaminosis D, the associations between vitamin D levels, bipolar disorder pathophysiology and cognitive features are not well understood.

The mechanism underlying vitamin D's influence on brain function is complex and likely to involve direct induction of neurotrophic factors and indirect anti-inflammatory and metabolic effects.^17^ Research suggests a connection between inflammation and the pathogenesis of bipolar disorder.^18^ In addition, inflammatory markers were identified, suggesting that inflammation could contribute to the neurocognitive deficits observed in bipolar disorder.^19,20^ Therefore, the anti-inflammatory neuroprotective role of vitamin D may preserve axonal integrity in the face of neuro-damage, which can be identified as neurofilament light chain (NfL) levels.^10,21^ The NfL is a unique biomarker related to axonal damage and neural cell death. It demonstrates promise as a potential marker of neuropsychiatric disease activity and progression associated with cognitive deficit.^22,23^ Patients with bipolar disorder have been reported to have higher NfL levels, both in their central nervous systems and peripheral blood, compared with healthy controls.^23,24^ However, studies analysing the association between vitamin D, NfLs and the cognitive profile of bipolar disorder have not been conducted.

## Aims of the study

We hypothesised that both vitamin D levels and NfLs have individual effects on cognition in bipolar disorder, with vitamin D indicating an anti-inflammatory modifiable nutrition status and NfLs indicating neuroaxonal damage over the disease course. Cognitive impairment in bipolar disorder may be neurodevelopmental, neuroprogressive or a combination of the two. Therefore, the aim of the study was to investigate the interaction of these two biomarkers in patients with bipolar disorder of different ages, to enable future studies to clarify the underlying mechanism of bipolar disorder cognition.

## Method

### Participants

In this cross-sectional study, we recruited 100 individuals who had been given a diagnosis of bipolar disorder type 1 in accordance with the DSM-5, from the out-patient clinic of a tertiary psychiatric hospital. The individuals were aged from 20 to 65 years and had not taken vitamin D supplements within 3 months of the study. We excluded individuals with (a) a known substance use disorder (with the exception of a nicotine use disorder); (b) any disorder with neurological symptoms or complications, such as brain injury or stroke; (c) an active physical illness such as renal impairment or hepatic failure, or pregnancy; (d) a previous diagnosis of intellectual disability, schizophrenia or schizoaffective disorders or (e) an inability to complete the standard clinical assessment or provide informed consent. The information regarding psychiatric comorbidities and the exclusion criteria was obtained through patient medical records and the Chinese version of the modified Schedule of Affective Disorder and Schizophrenia-Lifetime. Individuals were also required to be euthymic and under stable medication (no change in psychotropic medications in the previous month).

The authors assert that all procedures contributing to this work comply with the ethical standards of the relevant national and institutional committees on human experimentation and with the Helsinki Declaration of 1975, as revised in 2008. All procedures involving human patients were approved by the Research Ethics Committee of Taipei City Hospital (approval number TCHIRB-10912011). Written informed consent was obtained from all patients.

### Measurements

#### Demographic data, clinical course and mood symptoms

Patients’ demographic and clinical course data were collected from medical records and interviews with psychiatrists, if required. Participant clinical characteristics included number of affective episodes (total, manic and major depressive), number of episodes with psychotic features, number of hospital admissions and age at illness onset. Because the duration of illness varied across individuals, we calculated episode density by dividing the number of episodes by the duration of illness, to obtain the total episode density, manic episode density, major depressive episode density and episodes with psychotic features density. The psychopharmacological medications the patients used at the time of assessment were recorded and converted to a defined daily dose (DDD). A DDD is a unit of measurement representing the assumed average maintenance dose per day for a drug used for its main indication in adults, which can be used for comparisons of drug consumption. Mood symptoms were obtained through clinician-administered measures, that is, through the 17-item Hamilton Rating Scale for Depression (HRSD) and the Young Mania Rating Scale (YMRS). We defined euthymia as both HRSD and YMRS ≤8, screening within 1 week before the cognitive assessment.

#### Cognitive measurements

The enrolled patients were assessed with the Brief Assessment of Cognition in Affective Disorders (BAC-A), which has extensively been used as a quick and reliable cognitive assessment of patients with a wide range of clinical affective disorders.^25^ The BAC-A can be administered in approximately 35 min. The assessment measures affective memory and emotional inhibition through the Affective Auditory Verbal Learning Test and Emotional Stroop Task, and measures six standard neurocognitive domains, namely working memory (Digit Sequencing Task), motor speed (Token Motor Task), verbal fluency (Category Instances and Controlled Oral Word Association Test), processing speed (Symbol Coding), verbal memory (List Learning) and executive function (Tower of London), through a comparison with norm references.^26^ The criterion and construct validity for each test of cognitive impairment, as well as the sensitivity of these tests to changes in cognition, have been demonstrated in the literature. Each test has also been demonstrated to be valid for use in different cultures and language groups.^26^ Premorbid IQ was estimated through the Adult Reading Test from the Wechsler Adult Intelligence Scale, by a licensed psychologist.

#### Physical activity and dietary patterns

Physical activity and dietary patterns are key variables affecting both vitamin D levels and cognition. We used the Chinese version of the International Physical Activity Questionnaire, which was reported to be a valid and reliable self-reported assessment of physical activity,^27^ to assess participants’ regular physical activity levels. We used the food frequency questionnaire to assess participants’ basic dietary patterns because vitamin D levels may be influenced by dietary habits. The food frequency questionnaire can measure long-term dietary intake, which is essential information for understanding dietary composition.

#### Vitamin D and NfL level analysis

The patients underwent fasting blood sampling in the early morning on the same day of cognitive assessment. A venous blood sample of 10 mL was taken from each participant, and the serum was separated and stored at −80°C until analyses were conducted. Serum vitamin D levels, measured as 25-hydroxyvitamin D (25(OH)D), were determined by chemiluminescent immunoassay technology with a commercially available kit (LIAISON®, DiaSorin Inc., Stillwater, MN, USA). Serum vitamin D levels were considered deficient if the serum 25(OH)D values were <20 ng/mL.^28^ NfL levels were measured by Quanterix Simoa® assay (Quanterix Corp., Lexington, MA, USA), a digital immunoassay with a lower detection limit of 0.104 pg/mL, according to the manufacturer's instructions.

### Statistical analyses

We first tested the influence of age on cognitive outcomes and assessed the associations between age and vitamin D and NfL levels. We compared the demographic and clinical characteristics of the patients with bipolar disorder with and without a vitamin D deficiency, stratified by age, with a cut-off value of 45 years, to balance the sample size and age distribution of the two age strata. *χ*²- and Student *t-*tests were used for assessing categorical and continuous variables, respectively. The normality of the data was assessed with the Kolmogorov–Smirnov test. For nonnormally distributed data, we used the nonparametric Wilcoxon rank-sum test. General linear models adjusted for age and gender were used to determine the associations between vitamin D and NfLs, and their interaction effects on cognitive domains. Furthermore, general linear models with additional adjustment for years of education, psychotropic medication DDD and the variables that significantly differed between the groups with and without vitamin D deficiencies were used in a second model. We determined an interaction plot representing the interaction effect of vitamin D and NfL levels on cognition in each age group. All analyses were conducted with SAS software (version 9.4 for Windows; SAS Institute, Cary, NC, USA), and significance was set at *P* < 0.05.

## Results

### Patient characteristics and age effect

We recruited 100 patients (48 men and 52 women) with bipolar disorder who were in a euthymic state. The mean age was 47.49 years (s.d. 11.71), and the mean age at onset was 24.78 years (s.d. 9.79). The median and mean NfL levels were 9.26 and 11.10 pg/mL, respectively. The mean vitamin D level was 16.46 ng/nL, with 72% of the patients exhibiting vitamin D deficiency. A significant positive association was identified between age and NfL levels (*r* = 0.377, *P* < 0.001), and a nonsignificant negative association was identified between age and vitamin D levels (*r* = −0.63, *P* = 0.266; scatterplot in Supplementary Fig. 1 available at https://doi.org/10.1192/bjo.2022.608). Age also had a significant negative correlation with composite neurocognitive score (*r* = −0.031, *P* < 0.001) and the neurocognitive domains of verbal memory (*r* = −0.16, *P* = 0.019), working memory (*r* = −0.039, *P* < 0.001), processing speed (*r* = −0.031, *P* < 0.001) and executive function (*r* = −0.028, *P* = 0.001) as determined through the BAC-A. With a cut-off value of 45 years, significant differences were identified in the composite neurocognitive score, working memory and executive function scores between age subgroups (Supplementary Table 1).

### Comparison of vitamin D status

Table 1 presents the sociodemographic and clinical characteristics of the patients with bipolar disorder with and without vitamin D deficiencies, stratified by age. In the younger age group, 62.79% exhibited vitamin D deficiency; in the older age group, 78.95% exhibited vitamin D deficiency. No significant differences were identified in the disease course, amount of physical activity, NfL levels or DDD of the younger patients with and without vitamin D deficiency. In the older age group, we noted significantly higher numbers of hospital admissions in the patients with versus without vitamin D deficiency (8.49 ± 8.55 *v*. 2.92 ± 2.88; *P* = 0.031). Furthermore, the patients without vitamin D deficiency reported more physical activity than those with (1552.67 ± 429.54 *v*. 666.56 ± 603.16; *P* = 0.016). No differences were noted in the ages, premorbid estimated IQs, gender, marital or job statuses, smoking or alcohol use habits, nutrient supplement intakes or proportions of physical comorbidities of the patients with and without vitamin D deficiency in both age groups. Moreover, the differences in cognitive function in the patients with and without vitamin D deficiency and in those with higher and lower median NfL levels were nonsignificant for both age groups (Supplementary Tables 2 and 3).

**Table 1:** Sociodemographic and clinical characteristics of patients with bipolar disorder with and without vitamin D deficiency, stratified by age

	Age 20–45 years (*n* = 43)			Age 46–65 years (*n* = 57)		
	Vitamin D non-deficiency (*n* = 16)	Vitamin D deficiency (*n* = 27)	*P*-value	Vitamin D non-deficiency (*n* = 12)	Vitamin D deficiency (*n* = 45)	*P*-value
Age, years	37.06 (6.59)	33.37 (6.40)	0.078	57.00 (4.82)	54.91 (4.83)	0.189
Age at onset, years	19.00 (2.13)	19.64 (4.53)	0.546	31.42(13.17)	28.07 (10.28)	0.353
Education, years	13.63 (2.35)	14.00 (2.17)	0.606	13.17 (3.43)	12.37 (3.51)	0.489
Estimated premorbid IQ	91.67 (22.69)	100.13 (15.28)	0.323	104.67 (3.22)	89.00 (17.11)	0.144
Total episode density	0.41 (0.20)	0.53 (0.16)	0.066	0.43 (0.40)	0.39 (0.23)	0.722
Manic episode density	0.34 (0.21)	0.32 (0.18)	0.835	0.24 (0.23)	0.28 (0.19)	0.487
Depressive episode density	0.07 (0.22)	0.20 (0.20)	0.076	0.19 (0.25)	0.11 (0.20)	0.158
Psychotic episode density	0.15 (0.14)	0.21 (0.19)	0.364	0.09 (0.10)	0.21 (0.22)	0.082
Number of hospital admissions	5.88 (5.23)	4.48 (4.27)	0.356	2.92 (2.88)	8.49 (8.55)	0.031*
Body mass index	23.14 (4.67)	21.58 (4.24)	0.302	23.48 (2.82)	24.12 (4.54)	0.659
HRSD score	2.19 (2.16)	3.19 (2.47)	0.188	2.83 (2.73)	2.47 (1.74)	0.570
YMRS score	2.50 (2.50)	2.52 (2.05)	0.978	2.58 (2.58)	3.38 (2.35)	0.312
IPAQ, kilocalories/week	1879.70 (307.76)	1780.21 (642.16)	0.873	1552.67 (429.54)	666.56 (603.16)	0.016*
NfL, pg/mL	9.32 (6.45)	7.15 (3.11)	0.144	10.84 (4.42)	14.18 (10.08)	0.269
DDD						
First-generation antipsychotics	0.19 (0.55)	0.03 (0.12)	0.068	0.00 (0.00)	0.08 (0.23)	0.261
Second-generation antipsychotics	1.70 (1.77)	1.23 (1.52)	0.812	1.53 (1.99)	1.98 (2.43)	0.587
Mood stabilisers	0.75 (0.43)	0.56 (0.47)	0.203	0.56 (0.39)	0.55 (0.38)	0.941
Antidepressants	0.00 (0.00)	0.09 (0.20)	0.367	0.08 (0.19)	0.07 (0.19)	0.986
Benzodiazepine	0.47 (0.64)	0.37 (0.78)	0.636	0.47 (0.63)	0.58 (0.70)	0.644
All psychotropic medications	2.28 (1.63)	2.15 (1.45)	0.774	1.68 (0.93)	2.13 (1.22)	0.135
Gender, *n* (%)			0.324			0.073
Male	7 (43.75)	16 (59.26)		8 (66.67)	17 (37.78)	
Female	9 (56.25)	11 (40.74)		4 (33.33)	28 (62.22)	
Marriage, *n* (%)			0.636			0.319
Married or cohabiting	2 (12.50)	4 (14.81)		5 (41.67)	26 (58.33)	
Other	14 (87.50)	23 (84.18)		7 (58.33)	19 (41.67)	
Job, *n* (%)			0.154			0.573
Regular job	8 (50.00)	7 (35.00)		3 (25.00)	8 (17.78)	
Other	8 (50.00)	20 (65.00)		9 (75.00)	37 (82.22)	
Smoking, *n* (%)			0.145			0.265
No	10 (62.50)	22 (81.48)		8 (66.67)	33 (73.33)	
Ever but quit	0 (00.00)	0		2 (16.67)	3 (6.67)	
Persistent	6 (37.50)	5 (18.52)		2 (16.67)	9 (20.00)	
Alcohol use, *n* (%)			0.962			0.154
No	14 (87.50)	22 (81.48)		9 (75.00)	38 (84.44)	
Sometimes	1 (6.25)	4 (14.81)		1 (8.33)	5 (11.11)	
Persistent	1 (6.25)	1 (3.70)		2 (16.67)	2 (4.44)	
Physical comorbidity, *n* (%)			0.141			0.226
No	12 (75.00)	22 (81.48)		7 (58.33)	30 (66.67)	
Yes	4 (25.00)	5 (18.52)		5 (41.67)	15 (33.33)	
Other nutrient supplements, *n* (%)			0.865			0.902
No	13 (81.25)	24 (88.89)		10 (83.33)	38 (84.44)	
Yes	3 (18.75)	3 (11.11)		2 (16.67)	7 (15.56)	

HRSD, Hamilton Rating Scale for Depression; YMRS, Young Mania Rating Scale; IPAQ, International Physical Activity Questionnaire; NfL, neurofilament light chain; DDD, defined daily dose.

* *P* < 0.05.

### Interaction effect of vitamin D and NfL level on cognition

**Fig. 1.**
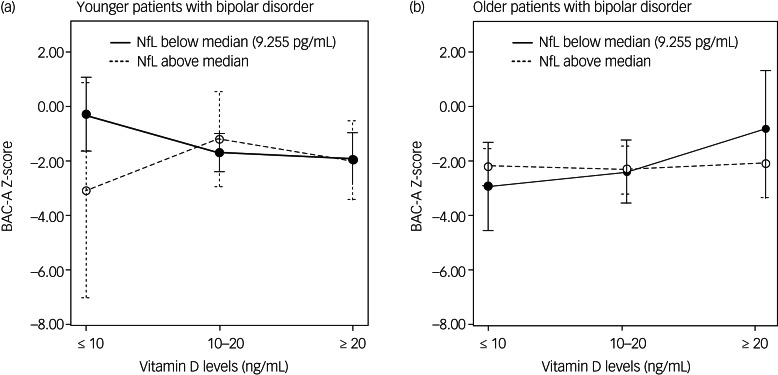
Interaction plots of effect of vitamin D and neurofilament light chain levels on cognition. (a) Younger patients with bipolar disorder and (b) older patients with bipolar disorder. BAC-A, Brief Assessment of Cognition in Affective Disorders; NfL, neurofilament light chain.

General linear models adjusted for age and gender, and models further adjusted for years of education, psychotropic DDD, number of hospital admissions and physical activity levels, were used to determine the effects of vitamin D, NfLs and their interaction on various cognitive domains in younger (Table 2) and older patients (Table 3) with bipolar disorder. In the younger age group, we identified a significant association between serum vitamin D, NfLs and their interaction to composite neurocognitive score. The association was particularly noted on verbal fluency and processing speed domains (model 2 in Table 2). In the younger age group, NfL levels had a considerable negative influence and vitamin D had a minor negative influence. Furthermore, the interaction of NfL and vitamin D levels positively affected cognitive function. In the older age group, the interaction between vitamin D and NfLs significantly and negatively affected composite neurocognitive score. The association was also particularly noted on verbal fluency (model 2 in Table 3). Moreover, vitamin D had a positive association with composite neurocognitive score; in addition, both vitamin D and NfLs had a significant positive association with verbal fluency in the older age group. In summary, we identified interaction effects of vitamin D and NfLs on composite neurocognitive function and verbal fluency domain for both age groups. Furthermore, age influenced the direction of the interaction effects. The crossed lines in the interaction plot (Fig. 1) indicate that the association between vitamin D status and cognition was influenced by NfL levels for both younger and older patients with bipolar disorder. In the younger patients with bipolar disorder with vitamin D levels <10 ng/mL, those with lower NfL levels exhibited more favourable cognitive function. However, when vitamin D levels were higher, the negative effect of NfLs was not present (Fig. 1(a)). In the older patients with bipolar disorder, the patients with lower NfL levels generally demonstrated increased cognitive function as their vitamin D status improved. However, the plot for the older patients with bipolar disorder with higher NfL levels revealed no notable cognitive changes in vitamin D levels (Fig. 1(b)).

**Table 2:** Associations between total serum vitamin D and neurofilament light chain and their interaction effect on cognitive domains in younger patients (aged 20–45 years) with bipolar disorder

		Model 1		Model 2	
		*ß* (95% CI)	*P*-value	*ß* (95% CI)	*P*-value
Composite score	Vitamin D	−0.121 (−0.262 to 0.019)	0.090	−0.344 (−0.671 to −0.016)	0.040*
	NfL	−0.200 (−0.583 to 0.183)	0.306	−0.574 (−1.144 to −0.005)	0.048*
	Vitamin D×NfL	0.010 (−0.007 to 0.028)	0.256	0.032 (0.001–0.062)	0.045*
Verbal memory	Vitamin D	−0.057 (−0.202 to 0.088)	0.442	−0.024 (−0.255 to 0.207)	0.838
	NfL	−0.102 (−0.497 to 0.294)	0.614	−0.067 (−0.469 to 0.334)	0.742
	Vitamin D×NfL	0.005 (−0.014 to 0.023)	0.630	0.030 (−0.019 to 0.025)	0.794
Motor speed	Vitamin D	−0.023 (−0.173 to 0.127)	0.767	−0.220 (−0.719 to 0.278)	0.386
	NfL	0.004 (−0.405 to 0.413)	0.983	−0.240 (−1.107 to 0.626)	0.587
	Vitamin D×NfL	0.001 (−0.018 to 0.020)	0.895	0.020 (−0.027 to 0.067)	0.408
Working memory	Vitamin D	−0.109 (−0.250 to 0.033)	0.132	−0.226 (−0.539 to 0.088)	0.159
	NfL	−0.139 (−0.524 to 0.246)	0.479	−0.419 (−0.966 to 0.126)	0.132
	Vitamin D×NfL	0.007 (−0.011 to 0.025)	0.455	0.019 (−0.010 to 0.050)	0.199
Verbal fluency	Vitamin D	−0.084 (−0.146 to −0.022)	0.008**	−0.230 (−0.347 to −0.113)	<0.001***
	NfL	−0.188 (−0.356 to −0.019)	0.029*	−0.522 (−0.726 to −0.319)	<0.001***
	Vitamin D×NfL	0.008 (0.001–0.016)	0.035*	0.024 (0.013–0.035)	<0.001***
Processing speed	Vitamin D	−0.156 (−0.295 to −0.017)	0.027*	−0.409 (−0.677 to −0.141)	0.003**
	NfL	−0.328 (−0.706 to 0.050)	0.089	−0.769 (−1.235 to −0.303)	0.001**
	Vitamin D×NfL	0.017 (−0.001 to 0.034)	0.063	0.041 (0.015–0.066)	0.002**
Executive function	Vitamin D	−0.014 (−0.135 to 0.107)	0.820	−0.168 (−0.576 to 0.240)	0.420
	NfL	0.027 (−0.304 to 0.357)	0.875	−0.111 (−0.821 to 0.598)	0.759
	Vitamin D×NfL	−0.001 (−0.016 to 0.015)	0.919	0.009 (−0.029 to 0.047)	0.647

Model 1: age, gender, vitamin D level, NfL level and interaction of vitamin D and NfLs. Model 2: age, gender, defined daily dose of total psychotropic medications, years of education, number hospital admissions, level of physical activity determined with the International Physical Activity Questionnaire, vitamin D level, NfL level and interaction of vitamin D and NfLs. NfL, NfL, neurofilament light chain.

* *P* < 0.05.

** *P* < 0.01.

*** *P* < 0.001.

**Table 3:** Association between total serum vitamin D and neurofilament light chain and their interaction effect on cognitive domains in older patients (aged 46–65 years) with bipolar disorder

		Model 1		Model 2	
		*ß* (95% CI)	*P*-value	*ß* (95% CI)	*P*-value
Composite score	Vitamin D	0.165 (0.046–0.284)	0.007**	0.347 (0.054–0.640)	0.020*
	NfL	0.074 (−0.017 to 0.166)	0.111	0.161 (−0.193 to 0.516)	0.373
	Vitamin D×NfL	−0.010 (−0.018 to −0.001)	0.023*	−0.027 (−0.049 to −0.004)	0.020*
Verbal memory	Vitamin D	0.120 (0.030–0.211)	0.009**	0.147 (−0.159 to 0.453)	0.347
	NfL	0.055 (−0.015 to 0.124)	0.122	0.089 (−0.281 to 0.460)	0.637
	Vitamin D×NfL	−0.006 (−0.013 to <−0.001)	0.047*	−0.013 (−0.037 to 0.011)	0.278
Motor speed	Vitamin D	0.121 (0.044–0.199)	0.002**	0.098 (−0.031 to 0.227)	0.317
	NfL	0.062 (0.003–0.121)	0.039*	−0.065 (−0.221 to 0.657)	0.418
	Vitamin D×NfL	−0.007 (−0.012 to −0.001)	0.018*	−0.003 (−0.013 to 0.007)	0.593
Working memory	Vitamin D	0.090 (−0.030 to 0.210)	0.142	0.123 (−0.206 to 0.452)	0.464
	NfL	0.027 (−0.065 to 0.119)	0.564	−0.080 (−0.478 to 0.318)	0.695
	Vitamin D×NfL	−0.005 (−0.014 to 0.003)	0.209	−0.008 (−0.034 to 0.017)	0.529
Verbal fluency	Vitamin D	0.049 (−0.004 to 0.102)	0.068	0.252 (0.114–0.390)	<0.001***
	NfL	0.014 (−0.027 to 0.054)	0.510	0.238 (0.071–0.404)	0.005**
	Vitamin D×NfL	−0.002 (−06006 to 0.001)	0.198	−0.019 (−0.030 to −0.009)	<0.001***
Processing speed	Vitamin D	0.121 (0.028–0.215)	0.011*	0.178 (−0.063 to 0.418)	0.147
	NfL	0.064 (−0.008 to 0.135)	0.080	0.066 (−0.226 to 0.357)	0.658
	Vitamin D×NfL	−0.007 (−0.014 to −0.001)	0.027*	−0.015 (−0.034 to 0.003)	0.108
Executive function	Vitamin D	0.098 (−0.033 to 0.228)	0.144	0.454 (−0.041 to 0.949)	0.072
	NfL	0.060 (−0.040 to 0.160)	0.240	0.302 (−0.298 to 0.902)	0.324
	Vitamin D×NfL	−0.008 (−0.017 to 0.001)	0.099	−0.038 (−0.076 to 0.001)	0.054

Model 1: age, gender, vitamin D level, NfL level and interaction of vitamin D and NfLs. Model 2: age, gender, defined daily dose of total psychotropic medications, years of education, number of hospital admissions, level of physical activity determined with the International Physical Activity Questionnaire, vitamin D level, NfL level and interaction of vitamin D and NfLs. NfL, neurofilament light chain.

* *P* < 0.05.

** *P* < 0.01.

*** *P* < 0.001.

## Discussion

### Main findings

In the younger patients with bipolar disorder, we noted a negative association between NfLs and vitamin D, and composite neurocognitive score and two neurocognitive domains (verbal fluency and processing speed). In the older patients, we noted a positive association between NfLs and vitamin D, and the cognitive domain of verbal fluency. Our results revealed a consistently significant antagonistic interaction effect on composite score and verbal fluency for both age groups. In addition, age was likely an effect-modified factor in the association. If NfLs can be regarded as a marker of neuroaxonal change over the course of bipolar disorder, clarifying the association of an intervening factor, such as vitamin D status, at different ages could considerably influence future intervention designs.

### Complex interaction of NfLs and vitamin D in bipolar disorder

**Fig. 2.**
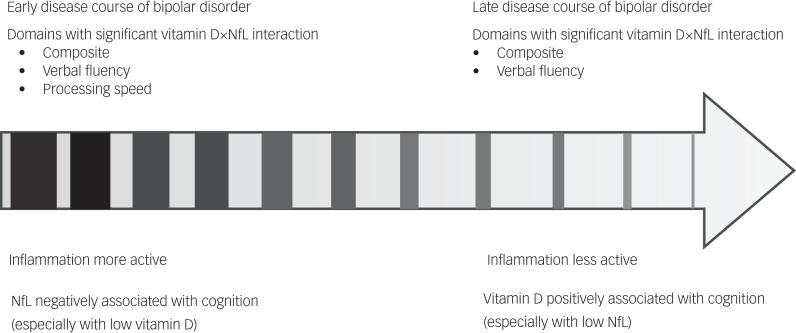
Inflammatory model and age-modified interaction effects in treated euthymic bipolar disorder. NfL, neurofilament light chain.

In interpreting the positive correlation between NfLs and verbal fluency in older patients with bipolar, researchers must consider that older patients have a longer disease course; therefore, early axonal damage could later have been affected by remyelination and axonal regeneration. This would be consistent with neuroimaging findings indicating that NfL level is positively associated with axial diffusivity in bipolar disorder.^24^ Our finding of a negative correlation between vitamin D and cognition in young patients with bipolar disorder contrasts with that of most studies on neuropsychiatric diseases.^29^ However, the parallel association (both negative or positive) identified between NfLs and vitamin D and cognition was consistent with the results of trials involving patients with multiple sclerosis, which has a similar neuropathology to bipolar disorder.^30,31^ In the aforementioned trials, disease status modified the association between vitamin D and NfL levels. In patients with active multiple sclerosis, the theoretical negative correlation of vitamin D and NfLs (non-parallel) have been reported to affect their brain function. However, in patients with multiple sclerosis in remittance or who received disease-modifying therapy, a parallel correlation or no correlation has been reported between vitamin D and NfLs and brain function.^32,33^ In this study, the patients with bipolar disorder were in a euthymic state and were receiving medication. This may explain the parallel association between vitamin D, NfLs and cognitive function in the two age groups, similar to the aforementioned multiple sclerosis findings. Our findings may further be explained by the following. First, in the early stages of the disease in younger patients with bipolar disorder, NfLs play a significant role in determining disease severity, which negatively influences cognition. Meanwhile, the influence of an individual's vitamin D status on cognition may be modified by other factors, such as medication. For example, although mood stabilisers are generally provided when treating bipolar disorder, they are frequently associated with reduced vitamin D levels.^34^ Second, in the older patients with bipolar disorder in our study, a positive association between vitamin D and cognitive function was identified, and vitamin D may enhance the remyelination process. This, together with the elevated NfL levels, led to a positive association between vitamin D and NfL levels and the verbal fluency domain. In addition, the age-modified interaction effect could contribute to evidence supporting the neuroimmune development of bipolar disorder, in which, at the onset of the illness, individuals experience an increased proinflammatory state in adolescence, an anti-inflammatory state during young adulthood and a nearly normalised immune state in later stages of the disease course.^35^ The interaction plot revealed an antagonistic interaction effect of vitamin D and NfLs, indicating that in the early stages of bipolar disorder in younger patients, NfL level may serve as a marker for neuro-damage resulting from neuroinflammation, and cognition differed in patients with higher and lower NfL levels, particularly those with extremely low vitamin D levels. In older patients with bipolar disorder who have a longer disease course, neuroinflammatory processes become stable, and the positive effects of vitamin D become more prominent in patients with lower rather than higher NfL levels. Therefore, our findings are consistent with the most current overview of the immune-inflammatory model in bipolar disorder,^36^ and further add the role of neuroaxonal damage and vitamin D status (Fig. 2).

### Possible causal inferences of the interaction

Studies involving animal models have reported effects of vitamin D on reversing neuro-cytoskeleton imbalances, but not memory deficits, and have reported the protective effects of vitamin D to be more favourable before and during, rather than after, the demyelination process.^37^ The cross-sectional design of this study prevented us from identifying any temporal association between vitamin D and NfLs. Therefore, we could not determine whether neuroinflammation with high NfL levels caused vitamin D depletion or vitamin D deficiency aggravated axonal abnormality over the disease course. In addition, some studies have suggested that the effects of vitamin D should be identified through vitamin D receptor polymorphisms rather than serum levels.^38^ Because vitamin D receptor polymorphisms also contribute to bipolar disorder, gene polymorphisms may influence both neuroaxonal integrity and the effects of vitamin D on brain function in bipolar disorder.^39^ Future longitudinal studies are required to investigate potential temporal associations between these factors. We also analysed the association between vitamin D, NfL levels and the interaction and clinical variables, such as clinical course and DDD, for each pharmacological category. We found only one positive association, between first-generation antipsychotic dose and NfL levels (data not shown). We need further studies to capture the whole picture of antipsychotics used during the disease course to understand the impact of antipsychotics on neuroaxonal integrity. Furthermore, few trials have investigated the influence of vitamin D levels on cognitive outcomes in psychiatric patients, with only one study involving patients with schizophrenia^40^ and one study on multiple sclerosis.^41^ Although both of the trials suggested a potential influence of vitamin D supplements on cognitive performance, the scarcity of studies on the subject prevents us from determining whether vitamin D can improve cognition in bipolar disorder. Our review of the literature revealed only two studies investigating the influence of vitamin D on bipolar disorder. One trial investigated bipolar depression with vitamin D deficiency, and revealed no impact of vitamin D intervention on depressive symptoms.^42^ Another open-label trial involving patients aged <18 years with bipolar disorder with mania revealed that vitamin D3 supplementation improved mood symptoms and brain neurochemistry.^43^ Neither trial included cognitive outcome measurements or changes in NfL levels. Further clinical trials focused on the influence of vitamin D intervention on cognitive outcomes in bipolar disorder and the influence of NfLs are required to verify our findings.

### Strengths and weaknesses

To our knowledge, this study is the first to provide evidence of the interaction effect of vitamin D and NfLs on neurocognitive domains in patients with bipolar disorder of different ages. Our study has several limitations. First, our study sample was recruited from a tertiary psychiatric hospital. Therefore, the patients likely had a more severe degree of illness and cognitive dysfunction. In addition, only the euthymic patients with bipolar disorder were recruited in this study, who were screened for their mood status and then referred for cognitive assessment within 1 week; therefore, some of the patients may be in early remission. This potential bias in our sample ascertainment may limit the generalisability of the current findings to the entire bipolar disorder population. Second, we do not have the longitudinal cognitive profile to ensure cognitive stability across episodes. Third, we did not have a healthy control to determine whether vitamin D deficiency or the age-modified interaction effect of NfL and vitamin D levels on cognitive function were unique to bipolar disorder. Fourth, the age restrictions we used in our sample limited our findings to older and teenaged patients with bipolar disorder. Additional bipolar disorder subgroups with new onset and longer disease duration should be included in future samples. Fifth, vitamin D status was assessed by measuring total serum 25(OH)D concentrations. However, only free (unbound) and albumin-bound vitamin D reflect biological activity;^44^ in addition, the effects of vitamin D should be identified through vitamin D receptor polymorphisms. Nevertheless, a study involving an Asian sample reported that total 25(OH)D concentrations were closely correlated with bioavailable 25(OH)D.^45^ Therefore, 25(OH)D can be regarded as a primary marker of vitamin D status. Sixth, peripheral NfL levels do not represent total NfL levels in the central nervous system. However, serum measurements of NfL levels have been considered reliable in many studies.^46^ Finally, as the cross-sectional study design, we precluded the causal effect of our findings, and further studies to clarify the linking mechanism are needed.

### Implications

We identified the interaction effect of vitamin D and NfLs on cognitive function in bipolar disorder. In addition, we discovered that age might play the role of an effect modifier. The effects of vitamin D on serum NfLs and cognitive outcomes in bipolar disorder warrant further study, with inclusion of patients with bipolar disorder with a range of mood statuses and disease courses. Longitudinal or randomised controlled trial designs are required to validate our findings and achieve more robust causal inferences and clinical implications.

## Data Availability

The data that support the findings of this study are available from the corresponding author, P.-H.K., upon reasonable request.

## Supplementary material

For supplementary material accompanying this paper visit http://doi.org/10.1192/bjo.2022.608. click here to view supplementary material
