# Supplementary material for: The interactions between vitamin D and neurofilament light chain levels on cognitive domains in bipolar disorder
Source: BJPsych Open. 2022 Nov 28;8(6):e207. doi: 10.1192/bjo.2022.608 (PMC9707506; doi:10.1192/bjo.2022.608)
Supplement: Supplementary file 1 [file S2056472422006081sup001.docx]

**Supplementary Figure 1.** Scatter plot for NfL and age, vitamin D levels and age


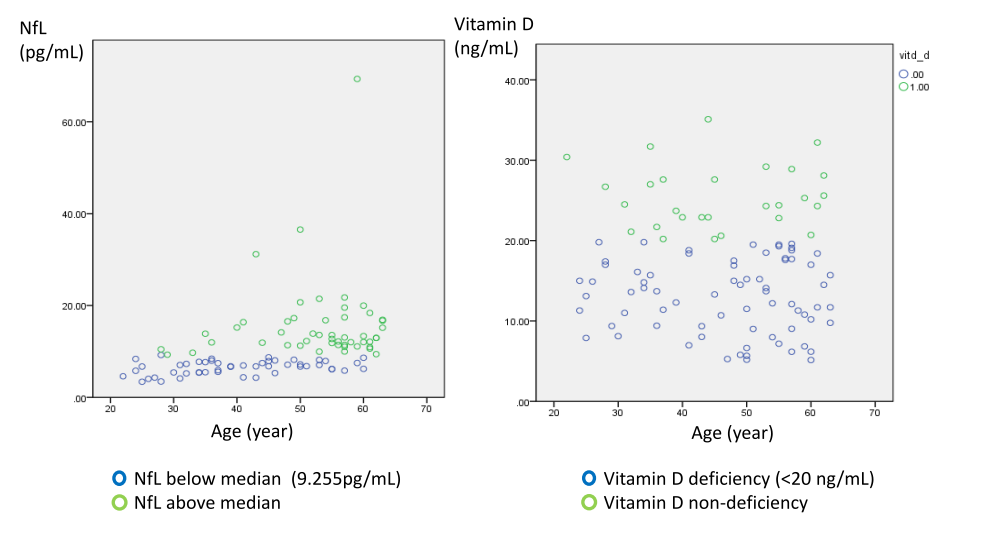


**Supplementary Table 1**. Correlations between BAC-A cognitive domains and age in patients with bipolar disorder

|  | BAC-A composite score | Verbal memory | Motor speed | Working memory | Verbal fluency | Processing speed | Executive function |
| --- | --- | --- | --- | --- | --- | --- | --- |
| age | r= -0.031  P< 0.001*** | r= -0.16  P= 0.019* | r= -0.001  P= 0.94 | r= -0.039  P< 0.001*** | r= 0.001  P=0.844 | r= -0.031  P<0.001*** | r= -0.028  P= 0.001** |
| P value between age groups (cutoff by 45 y/o) | 0.042* | 0.259 | 0.498 | 0.012* | 0.185 | 0.056 | 0.007** |

**Supplementary Table 2.** Comparison of cognitive domains between patients with bipolar disorder with and without vitamin D deficiency, stratified by age

|  | 20 ≤Age ≤45 (N=43) | | | 45< Age <65 (N=57) | | |
| --- | --- | --- | --- | --- | --- | --- |
|  | Vitamin D  Non-deficiency  (N =16 ) | Vitamin D  deficiency  (N = 27) | P-value | Vitamin D  Non-deficiency  (N =12 ) | Vitamin D  deficiency  (N = 45) | P-value |
| BAC-A composite score | -1.93 (1.34) | -1.48 (1.36) | 0.303 | -1.65 (1.49) | -2.35 (1.62) | 0.183 |
| Verbal memory | -1.47 (1.41) | -1.37 (1.35) | 0.811 | -1.28 (1.13) | -1.65 (1.23) | 0.351 |
| Motor speed | -0.62 (1.27) | -0.86 (1.48) | 0.597 | -0.35 (1.02) | -0.92 (1.03) | 0.096 |
| Working memory | -1.00 (1.57) | -0.23 (1.19) | 0.103 | -0.95 (1.35) | -1.31 (1.61) | 0.482 |
| Verbal fluency | -2.34 (0.47) | -1.98 (0.67) | 0.054 | -1.62 (0.76) | -2.04 (0.66) | 0.061 |
| Processing speed | -1.37 (1.37) | -1.08 (1.37) | 0.512 | -1.20 (1.01) | -1.73 (1.28) | 0.188 |
| Executive function | -0.44 (1.15) | -0.15 (1.13) | 0.422 | -0.91 (1.84) | -1.00 (1.66) | 0.867 |

**Supplementary Table 3.** Comparison of cognitive domains between patients with bipolar disorder with higher and lower NfL levels, stratified by age

|  | 20 ≤Age ≤45 (N=43) | | | 45< Age <65 (N=57) | | |
| --- | --- | --- | --- | --- | --- | --- |
|  | Lower NfL  (<9.255 pg/mL)  N=34 | Higher NfL  (>9.255 pg/mL)  N= 9 | P-value | Lower NfL  (<9.255 pg/mL)  N= 16 | Higher NfL  (>9.255 pg/mL)  N= 41 | P-value |
| BAC-A composite score | -1.56 (1.44) | -1.96 (0.99) | 0.450 | -2.12 (1.45) | -2.23 (1.68) | 0.814 |
| Verbal memory | -1.33 (1.48) | -1.69 (0.72) | 0.486 | -1.56 (1.14) | -1.58 (1.25) | 0.945 |
| Motor speed | -0.84 (1.50) | -0.51 (0.97) | 0.540 | -0.62 (1.08) | -0.87 (1.04) | 0.426 |
| Working memory | -0.41 (1.40) | -0.90 (1.32) | 0.339 | -1.23 (1.62) | -1.23 (1.54) | 0.996 |
| Verbal fluency | -2.05 (0.63) | -1.13 (1.44) | 0.372 | -1.86 (0.61) | -1.99 (0.73) | 0.530 |
| Processing speed | -1.13 (1.44) | -1.44 (1.01) | 0.546 | -1.63 (1.23) | -1.61 (1.26) | 0.952 |
| Executive function | -0.23 (1.16) | -0.36 (1.10) | 0.762 | -1.04 (1.26) | -0.96 (1.83) | 0.874 |
